# Labour market attachment dynamics in patients with concussion: a Danish nationwide register-based cohort study

**DOI:** 10.1186/s12889-023-17364-2

**Published:** 2023-12-13

**Authors:** Heidi Jeannet Graff, Volkert Siersma, Anne Møller, Frans Boch Waldorff, Frederikke Agerbo Modin, Hana Malá Rytter

**Affiliations:** 1 Danish Concussion Center, Copenhagen, Amagerfælledvej 56A, Copenhagen, 2300 Denmark; 2 The Research Unit for General Practice and Section of General Practice, Department of Public Health, University of Copenhagen, Øster Farimagsgade 5, Copenhagen, 1014 Denmark; 3 Psychiatric Center Ballerup, Mental Health Service of the Capital Region of Denmark, Center for Eating and Feeding Disorders Research (CEDaR), Maglevænget 32, Ballerup, 2750 Denmark; 4 Department of Psychology, University of Copenhagen, Øster Farimagsgade 2A, Copenhagen, 1353 Denmark; 5 Department of Neurology, University Hospital Bispebjerg - Frederiksberg, Nielsine Nielsens Vej 7, Copenhagen, 2400 Denmark

**Keywords:** Concussion, Mild traumatic brain injury, Post-concussive symptoms, Employment, Sickness absence, Unemployment, Reduced work ability, Disability pension, Retirement

## Abstract

**Background:**

Concussion may lead to persisting post-concussive symptoms affecting work ability and employment. This study examined the transitions between labour market states an individual can experience after the acute phase of concussion. The aim was to describe the incidence of favourable and adverse transitions between different labour market states (e.g., employment, sick leave) in relation to socioeconomic and health characteristics in individuals with concussion relative to matched controls.

**Methods:**

This Danish nationwide register-based cohort study extracted 18–60-year-old individuals between 2003–2007 with concussion from the Danish National Patient Register (ICD-10 S06.0). Controls were matched on age, sex, and municipality. Patients and controls were followed for 5 years starting three months after injury.

Exclusion criteria were neurological injuries and unavailability to the labour market in the inclusion period (2003–2007) and 5-years before injury (1998–2002). Labour market states were defined from transfer income data in the Danish Register for Evaluation of Marginalization. Incidence rates of transitions between these labour market states were analysed in multistate models. Transitions were bundled in favourable and adverse transitions between labour market states and the difference in incidence rates between individuals with concussion relative to matched controls were assessed with hazard ratios from Cox regression models.

**Results:**

Persons with concussion (*n* = 15.580) had a lower incidence of favourable transitions (HR 0.88, CI 0.86–0.90) and a higher incidence of adverse transitions (HR 1.30, CI 1.27–1.35), relative to matched controls (*n* = 16.377).

The effect of concussion differed depending on health and socioeconomic characteristics. Notably, individuals between 30–39 years (HR 0.83, CI 0.79–0.87), individuals with high-income (200.000–300.000 DKK) (HR 0.83, CI 0.80–0.87), and wage earners with management experience (HR 0.60, CI 0.44–0.81) had a markedly lower incidence of favourable transitions compared to controls. Additionally, individuals with high income also had a higher incidence of adverse transitions (HR 1.46, CI 1.34–1.58) compared to controls.

**Conclusions:**

Concussion was associated with enhanced risk of adverse transitions between labour market states and lower occurrence of favourable transitions, indicating work disability, potentially due to persistent post-concussive symptoms. Some age groups, individuals with high income, and employees with management experience may be more affected.

**Supplementary Information:**

The online version contains supplementary material available at 10.1186/s12889-023-17364-2.

## Background

Mild traumatic brain injury, often denoted as concussion, is the most common form of all traumatic brain injuries [1]. Worldwide incidence rates on hospital-treated concussion for the years 1980–2000 range from 100 to 800 per 100.000 people [2]. Danish hospital-incidence rates for 1996 are about 400 per 100.000 people [3]. A recent study including both hospital- and general practice treated individuals reports up to approximately 1150 per 100.000 people [4]. The heterogeneity in the incidence rates is mostly due to differing methodological approaches, differences in diagnostics, health care infrastructure and health care seeking behaviour [5].

Persisting post-concussive symptoms (PCS) present in 30–40% of individuals with concussion up to 12 months post-injury or even longer [6–8]. PCS refer to a range of physical, cognitive, emotional, and behavioural symptoms. PCS is known to be associated with long-term decreased quality of life, increased levels of anxiety and depression [9] and occupational limitations up to several years post-injury with a higher occurrence of long-term sickness absence and work disability [10–12].

Although most concussed individuals return to work within the first six months post-injury, individuals with concussion have an increased risk of early exclusion from the labour market [12]. Subgroups with concussion at risk of not attending ordinary work (ordinary work is defined as the employee receiving a salary from the employer and where the employer does not receive a subsidy for the salary) five years after the concussion in comparison to the background population, are individuals with higher education, adults between 30–39 years, individuals from ethnic minorities and individuals diagnosed with somatic comorbidities [13].

Studies have attempted to investigate the impact of concussion on employment in various ways. Some used the average time for patients to return to work (RTW) and the proportion of RTW at different time points following concussion [12]. Others examined the proportion of patients not returning to ordinary work focusing on different social transfer payments, e.g., sickness absence and disability pension [10]. Some studies focused on the quality of RTW defined as stable employment at several follow-up intervals, unstable employment, indicated as RTW to only a limited degree, and unemployment, indicated as no RTW [14, 15]. Research that only examines the average time off work, the proportion of individuals returning to work at different time points or a single state in the labour market trajectory such as unemployment due to e.g., sickness absence, falls short in describing the many different transitions between possible labour market states an individual with concussion can experience. The rates of transitions between various states such as sickness absence, work, unemployment, flex job and disability pension can be examined in multistate models. Such models focus on which transitions have the highest incidence and identify possible socioeconomic or health-related factors affecting the incidence of the various transitions [16]. Multistate models have previously been used to assess employment in other patient groups [17, 18], but have not been used to assess the long-term effect of concussion. This study seeks to expand the knowledge of the occupational consequences of concussion beyond the early post-injury phase by using this method.

Based on previous research we hypothesise that there will be a considerable number of individuals with PCS and that the ability to work will be adversely affected well beyond the subacute phase of injury in individuals with concussion. This will translate into a higher incidence of adverse labour market transitions (e.g., transitioning from work to sickness absence, flex job or disability pension) and possibly a lower incidence of favourable transitions (e.g., transitioning from sickness absence to work) of this group compared to matched controls.

Hence, the aim of the present study was to describe the incidence of favourable and adverse labour market transitions in patients with concussion relative to matched controls in relation to socioeconomic and health-related characteristics.

## Methods

This is a nationwide register-based cohort study following working age adults with concussion regarding transitions across different labour market states over a 5-year period after the time of injury. Data was extracted from national administrative registers provided by Statistics Denmark, using the unique personal identification number, the central personal registry number (CPR number) from the Danish Civil Registration System (CRS) [19].

### Study population and data sources

The study population was extracted from the Danish National Patient Register (DNPR), which contains information on in- and outpatients and emergency treated contacts in Danish secondary care registered with International Classification of Diseases, version 10 (ICD-10) diagnosis codes [20].

The study population consisted of people living in Denmark between 18–60 years who in the inclusion period from the 1^st^ of January 2003 to the 31^st^ of December 2007 were hospital admitted, i.e., had an emergency department visit or an outpatient visit in a Danish hospital with a primary diagnosis of concussion (an entry in the DNPR with ICD-10 code S06.0). People with more than one concussion admission in the inclusion period were included with the first concussion admission.

From this study population we excluded subjects with an admission to a Danish hospital with a primary or secondary diagnoses of neurological injuries such as spinal cord injuries and more severe traumatic brain injuries than concussion in the inclusion period. Additionally, we excluded subjects when they had these diagnoses, now including concussion, from the 5-year period from the 1^st^ of January 1998 to the 31^st^ of December 2002, before the inclusion period (See Additional file 1). This was to prevent confounding effects of other injuries on the associations of interest.

Subjects were further included if they were employed, in education or had temporary leave of absence from work (e.g., students and maternity leave). Hence, subjects were excluded, if they were unavailable to the labour market, defined as receiving any municipal granted social transfer payments e.g., due to unemployment, sick leave, disability pension, or retirement at their index date, i.e., one week before the first hospital admission with concussion with data from the Danish Register for Evaluation of Marginalization (DREAM) [21].

For each of the subjects with concussion one control subject without concussion in the inclusion period was added to the study population at the index date. Control subjects were sampled from the Population register which includes data from CRS [19] and matched on sex, municipality, and age (year of birth ± 0.5 years). The same exclusion criteria were used to determine the eligible controls for each subject with concussion as were used for the selection of subjects with concussion, including severe neurological injuries and concussion in the inclusion period and the 5-year period before the index date and being unavailable to the labour market. The index date for the control subject was the same as for its matched subject with concussion.

### Outcome measures

Labour market outcomes were defined with weekly updated data from DREAM, which covers all residents receiving municipal granted social transfer payments from the state, which are administered by the Danish ministries of Employments, Social Affairs and Education [21]. The study subjects were followed-up for a maximum of five years after the index date. We defined seven different labour market states: *Employment*, *Unemployment*, *Limited attachment to the labour market*, *Sick leave*, *Disability pension*, *Temporary leave*, and *Retirement*. These were augmented with three states not directly related to the labour market: *Emigration*, *Death* and *End-of-Follow-up (EoF)* (automatically reached five years after index date). The subjects in the study population traverse through these states until one of the five absorbing states, i.e., states indicating a permanent leave from the labour market or registration in DREAM, is reached: *Disability pension*, *Retirement*, *Emigration*, *Death*, or *EoF*. Consequently, subjects already in one of these absorbing states at the start of the registration are excluded from the analyses.

Registration of the states and transitions between them started three months after each subject’s index date and proceeded until an absorbing state was reached. The three-month run-in period was chosen to exclude acute consequences of the concussion (e.g., the sick leave immediately after concussion), and is based on the Diagnostic and Statistical Manual of Mental Disorders, Fourth Edition (DSM-IV), which states that symptoms extending beyond the period of three months should be designated PCS [22].

Employment was defined as being at work and self-supporting and not receiving any social transfer payments registered in DREAM during follow-up. Temporary leave included maternity leave, sabbatical leave, and leave from work with or without State education grant, including students. An unemployment state was indicated as being available to the labour market and actively job seeking receiving unemployment benefits or social security benefits [23]. Unemployed individuals were granted economic compensation (unemployment benefits), if they were actively job seeking for a maximum of four years [23]. After four years it was possible to receive social security benefits [23]. Sick leave was indicated by receiving sickness absence benefits [23]. Sickness benefits could be granted for a maximum of 52 weeks within a period of 18 months [24]. Limited attachment to the labour market was defined as a state of work disability without possibilities to maintain gainful employment. In this state patients with concussion were employed or available to the labour market under special circumstances, e.g., receiving flex job or vocational rehabilitation [23]. The state of disability pension was only granted to persons with a permanent lack of work ability resulting in an exclusion from the labour market [25]. Retirement was indicated by transitional allowance (voluntary withdrawal from the labour market before retirement age), state retirement pension at 65 years, early retirement (voluntary withdrawal from the labour market before retirement age for individuals who had paid for such a scheme) and retirement under the flex job scheme. Disability pension and retirement are absorbing states, due to exclusion from the labour market.

Transitions were bundled into: (1) Favourable transitions indicated those who returned to gainful employment after unemployment, a period of sickness absence or other social benefits indicating reduced work ability (e.g., flex job); (2) Adverse transitions were all transitions from gainful employment to absence due to sickness, unemployment, etc. Transitions between states within these bundles, e.g., from employment to temporary leave, were not regarded as transitions in this aggregation.

A brief description of the states is shown in Table 2 listing the study’s main results.

### Covariates

Data on age, sex and place of birth were extracted from the Population register [25]. Information on cohabitation status was obtained from the Danish Family Relations Database which is a part of the Population register. Preinjury somatic comorbidities were assessed with Charlson’s comorbidity index (CCI) [26]; a weighted sum of the occurrence of 19 diagnosis groups in the DNPR in the 5 years preceding the index date (1998–2002) and dichotomised into ≥ 1 comorbidities or none. Preinjury psychiatric comorbidities were assessed as a registration of a psychiatric diagnosis (ICD-10 F diagnosis) in the DNPR.

Education was measured as the highest attained educational level from the Danish Education Register. Preinjury income was obtained from the Income Statistics Register and measured as the personal gross income including revenue and social transfer income. Finally, we included data on employment status from the Employment Classification Module, which contains information on employment activity or the most important source of income. This makes it possible to determine whether the person is self-employed, wage earner, wage earner with management experience or not employed. Data is based on tax-reported income registers as an aggregate measurement of the income received for the whole year before the index date. Not employed is categorised as temporary leave of absence from work, in education and unemployment (e.g., unemployment benefits or social security benefits) [27].

## Statistical analysis

The differences between covariates between subjects with concussion and their matched controls were assessed with chi-squared tests.

Multi-state models describe the incidences of transitions between states using a survival model where the observation for an individual is the time until the occurence of the event, described as a transiton from an origin state to a target state (e.g., the transition from employment to sick leave) [28]. In the concussion and control cohort, we separately calculated the incidence rates for the 45 potential transitions from one of the five non-absorbing origin states to one of the ten target states. These rates were determined by dividing the number of such transitions by the total time spent in the origin state. A confidence interval was calculated using a Poisson assumption. Only transitions related to the labour market are reported. For adverse and favourable transitions, the ratio of the incidence rates of patients with concussion relative to their matched controls, was assessed by a hazard ratio (HR) from a Cox regression model where matching and recurrent transitions per subject in the study is adjusted for by a robust sandwich estimator [29]. In all these analyses, transitions into disability pension, retirement, emigration, death, or EoF are absorbing. Each analysis in the multistate model was controlled for possible confounding by adjusting for the nine covariates. *P*-values < 0.05 were regarded as statistically significant. SAS V.9.4 was used for statistical analysis.

## Results

### Social and health characteristics of patients with concussion and matched controls

Table 1 describes the social and health characteristics of individuals with concussion (*n* = 15.580) and matched controls (*n* = 16.377).

**Table 1:** Baseline characteristics of individuals with concussion and matched controls assessed at the index date

	**Concussion** (*n* = 15.580)	**Controls** (*n* = 16.377)	**Total** (*n* = 31.957)	**Missing**	***p*-value**
**Age**, *n (%)*				0 (0.0)	< .0001
18–29 years	7366 (47.3)	7344 (44.8)	14710 (46.0)		
30–39 years	3204 (20.6)	3558 (21.7)	6762 (21.2)		
40–49 years	2701 (17.3)	3036 (18.5)	5737 (18.0)		
50–60 years	2309 (14.8)	2439 (14.9)	4748 (14.9)		
**Sex,** *n (%)*				0 (0.0)	0.0320
Men	9342 (60.0)	10012 (61.1)	19354 (60.6)		
Women	6238 (40.0)	6365 (38.9)	12603 (39.4)		
**Ethnic origin,** *n (%)*				351 (0.9)	0.4963
Danish born	14797 (95.9)	15527 (96.0)	30324 (95.9)		
Born abroad	638 (4.1)	644 (4.0)	1282 (4.1)		
**Cohabitation status,** *n (%)*				54 (0.1)	< .0001
Married or cohabiting	5636 (36.2)	4711 (28.9)	10347 (32.4)		
Single	9938 (63.8)	11,618 (71.1)	21556 (67.6)		
**Charlson’s Comorbidity Index (CCI),** *n (%)*				0 (0.0)	< .0001
No comorbidities	14797 (95.0)	15769 (96.3)	30566 (95.6)		
≥ 1 comorbidity	783 (5.0)	608 (3.7)	1391 (4.4)		
**Psychiatric diagnosis,** *n (%)*				0 (0.0)	< .0001
No diagnosis	15003 (96.3)	16164 (98.7)	31167 (97.5)		
≥ 1 diagnosis	577 (3.7)	213 (1.3)	790 (2.5)		
**Education**^**a**^**,*** n (%)*				0 (0.0)	< .0001
Low education	6605 (42.4)	5891 (36.0)	12496 (39.1)		
Medium education	6213 (39.9)	7154 (43.7)	13367 (41.8)		
High education	2604 (16.7)	3169 (19.4)	5773 (18.1)		
**Income (Danish kroner, DKK),** *n (%)*				29 (0.1)	< .0001
< 100,000	3484 (22.4)	3663 (22.4)	7147 (22.4)		
100,000–200,000	3451 (22.2)	3047 (18.6)	6498 (20.4)		
200,000–300,000	4761 (30.6)	4860 (29.7)	9621 (30.1)		
> 300,000	3882 (24.9)	4780 (29.2)	8662 (27.1)		
**Pre-injury employment status,** *n (%)*				27 (0.1)	< .0001
Self employed	654 (4.2)	738 (4.5)	1392 (4.4)		
Wage earners with management experience	189 (1.2)	319 (2.0)	508 (1.6)		
Wage earners without management experience	10482 (67.3)	10908 (66.7)	21390 (67.0)		
Not employed^b^	4254 (27.3)	4386 (26.8)	8640 (27.1)		

^a^Low education (primary education), medium education (lower and upper secondary education, post-secondary–non-tertiary education) and high education (short-cycle tertiary education, bachelor, master, doctoral or equivalent

^b^Not employed includes temporary leave of absence from work (e.g., maternity leave), being in education and unemployment (e.g., unemployment benefits or social security benefits) as an aggregate measure of employment status for the whole year before the index date

Individuals with concussion were disproportionally more often married or cohabiting, having more comorbidities and psychiatric diagnosis, lower educated and with a lower income, and having a pre-injury employment status indicated by less management experience, relative to controls in our sample.

### The incidence of labour market transitions

Table 2 shows the crude incidence per 100 person years of the various transitions between the seven different labour market states for individuals with concussion and matched controls expressed with an IR and a 95% CI. Table 2 describes the labour market transitions from the state indicated in the left row to the state indicated in the top column and marked as favourable (green), adverse transitions (yellow), or transitions within these two bundles (grey). As an example, patients with concussion had an incidence of transitioning from employment to sick leave of 30.9 (95% CI 29.8–32.0), which is somewhat higher than the corresponding incidence of their matched controls (crude IR 19.0, 95% CI 18.2–19.9). Transitions not related to the labour market (emigration, death and EoF) are not included.

**Table 2:**
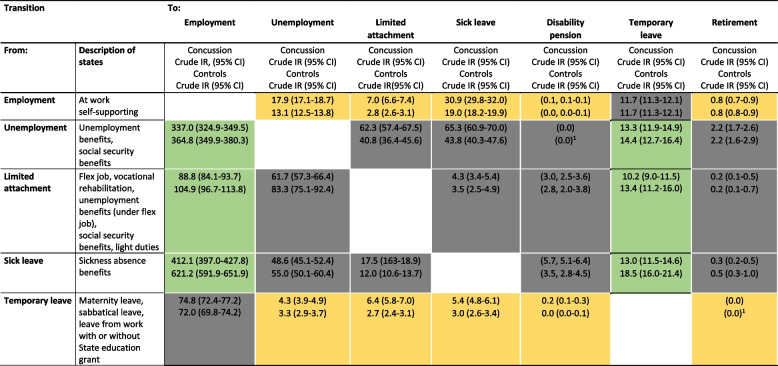
Incidence rates (IR), in 100 patient years, for the transitions between labour market states separately for individuals with concussion and matched controls

^a^These labour market transitions are legally unattainable [23, 24]

### Favourable transitions between labour market states

The favourable transitions (marked green in Table 2) showed a tendency towards a higher incidence for matched controls, where transitioning from sick leave to employment was associated with the highest incidence compared to individuals with concussion. The incidence of transitioning from unemployment to employment, respectively, and from limited attachment to the labour market (flex job) to employment was also higher for matched controls compared to individuals with concussion.

### Adverse transitions between labour market states

Of all the possible adverse transitions (marked yellow in Table 2), the highest incidence was found for individuals with concussion experiencing the transition from employment to sick leave compared to controls. Additionally, individuals with concussion had the highest incidence for the transition from employment to unemployment compared to matched controls.

There was also a higher incidence of transitioning from employment to limited attachment to the labour market and from temporary leave to limited attachment to the labour market and sick leave among individuals with concussion compared to controls.

### Labour market transitions according to socioeconomic and health related characteristics

The crude and adjusted HR for favourable and adverse transitions between labour market states for individuals with concussion relative to matched controls according to socioeconomic and health-related characteristics are shown in Table 3. Only results with a *p* < 0.05 for the interaction between subgroups are reported in the result section. Additional results can be seen in Table 3.

**Table 3:** The ratio of the incidence rates (hazard ratio; HR) of the favourable and adverse transitions between patients with concussion and their matched controls by socioeconomic and health characteristics

	**Favourable transitions**				**Adverse transitions**			
	Crude HR (95% CI)	*p*-value interaction	Adjusted HR^a^ (95% CI)	*p*-value interaction	Crude HR (95% CI)	*p*-value interaction	Adjusted HR^a^ (95% CI)	*p*-value interaction
**Total**	0.86 (0.84–0.88)		0.88 (0.86–0.90)		1.38 (1.34–1.43)		1.30 81.27–1.35)	
**Age**		0.0007		0.0066		0.1986		0.7807
18–29 years	0.90 (0.87–0.93)		0.91 (0.88–0.94)		1.34 (1.29–1.39)		1.30 (81.25–1.35)	
30–39 years	0.80 (0.76–0.85)		0.83 (0.79–0.87)		1.46 (1.37–1.56)		1.34 (1.26–1.44)	
40–49 years	0.82 (0.77–0.87)		0.84 (0.79–0.89)		1.36 (1.25–1.49)		1.28 (1.18–1.39)	
50–60 years	0.85 (0.79–0.91)		0.87 (0.81–0.94)		1.36 (1.23–1.50)		1.31 (1.18–1.44)	
**Sex**		0.43		0.5797		0.0035		0.1546
Men	0.87 (0.84–0.89)		0.88 (0.85–0.91)		1.43 (1.38–1.49)		1.33 (1.28–1.38)	
Women	0.85 (0.82–0.88)		0.87 (0.84–0.90)		1.31 (1.24–1.37)		1.27 (1.21–1.33)	
**Ethnic origin**		0.2738		0.3395		0.2549		0.5621
Born in Denmark	0.86 (0.84–0.88)		0.88 (0.86–0.90)		1.39 (1.34–1.43)		1.31 (1.27–1.35)	
Not born in Denmark	0.81 (0.73–0.90)		0.83 (0.75–0.92)		1.29 (1.14–1.46)		1.26 (1.11–1.42)	
**Cohabitation status**		0.3192		0.4756		0.2901		0.335
Married or cohabiting couple	0.86 (0.83–0.89)		0.87 (0.84–0.90)		1.38 (1.33–1.44)		1.32 (1.27–1.37)	
Single	0.88 (0.85–0.91)		0.89 (0.85–0.92)		1.34 (1.27–1.40)		1.28 (1.22–1.34)	
**CCI (categorical)**		0.6653		0.8015		0.7542		0.8528
No comorbidities	0.84 (0.75–0.95)		0.86 (0.77–0.97)		1.41 (1.23–1.62)		1.32 (1.15–1.52)	
≥ 1 comorbidity	0.86 (0.84–0.88)		0.88 (0.86–0.90)		1.38 (1.34–1.42)		1.30 (1.26–1.35)	
**Psychiatric diagnosis**		0.3647		0.3874		0.6883		0.7281
No diagnosis	0.92 (0.80–1.06)		0.93 (0.81–1.07)		1.42 (1.19–1.68)		1.35 (1.12–1.61)	
≥ 1 diagnosis	0.86 (0.84–0.88)		0.87 (0.85–0.89)		1.37 (1.32–1.41)		1.30 (1.26–1.34)	
**Education**^**b**^		0.1837		0.6498		0.0883		0.2619
Low education	0.88 (0.85–0.91)		0.89 (0.86–0.92)		1.32 (1.27–1.38)		1.28 (1.23–1.33)	
Medium education	0.85 (0.81–0.88)		0.86 (0.83–0.90)		1.35 (1.28–1.42)		1.30 (1.24–1.37)	
High education	0.86 (0.80–0.91)		0.87 (0.82–0.93)		1.48 (1.33–1.63)		1.43 (1.29–1.58)	
**Income (DKK)**		0.005		0.0029		0.0152		0.0229
< 100,000	0.91 (0.87–0.96)		0.92 (0.88–0.97)		1.34 (1.27–1.41)		1.30 (1.23–1.38)	
100,000–200,000	0.89 (0.85–0.93)		0.91 (0.87–0.95)		1.34 (1.28–1.42)		1.29 (1.22–1.36)	
200,000–300,000	0.82 (0.79–0.86)		0.83 (0.80–0.87)		1.28 (1.21–1.35)		1.25 (1.18–1.32)	
> 300,000	0.85 (0.80–0.90)		0.87 (0.82–0.92)		1.51 (1.39–1.65)		1.46 (1.34–1.58)	
**Pre-injury employment**		0.0065		0.0027		0.2862		0.1113
Self employed	0.75 (0.66–0.85)		0.76, 0.67–0.87)		1.21 (1.03–1.42)		1.11 (0.95–1.31)	
Wage earners with management experience	0.60 (0.45–0.81)		0.60 (0.44–0.81)		1.50 (1.00–2.24)		1.39 (0.94–2.06)	
Wage earners without management experience	0.71 (0.61–0.81)		0.71 (0.62–0.82)		1.21 (1.02–1.45)		1.09 (0.92–1.30)	

^a^The adjusted HR is adjusted by age, sex, place of birth, cohabitation status, somatic and psychiatric comorbidities, education, income, and employment status

^b^low education (primary education), medium education (lower and upper secondary education, post-secondary–non-tertiary education) and high education (short-cycle tertiary education, bachelor, master, doctoral or equivalent)

The total adjusted HR shows that individuals with concussion have a lower incidence of favourable transitions such as transitioning from sick leave or other social benefits to employment (HR 0.88, CI 0.86–0.90) compared to controls. Individuals with concussion also tend to have a higher incidence of adverse transitions (HR 1.30, CI 1.27–1.35), e.g., from employment to absence due to sickness, unemployment, etc. relative to matched controls.

Of the four age groups, individuals with concussion between 30–39 years had the lowest incidence of favourable transitions (HR 0.83, CI 0.79–0.87), closely followed by individuals between 40–49 years relative to matched controls. There was no significant difference in HR for adverse transitions between age groups. However, age group between 30–39-years had the highest HR for adverse transitions, which could indicate that these age groups are most affected on labour market attachment.

Individuals in the two highest income categories (200,000–300,000 DKK and > 300,000 DKK) presented with the strongest effect of concussion on favourable transitions (HR 0.83, CI 0.80–0.87) (HR 0.87, CI 0.82–0.92), respectively, and individuals with high income (> 300,000 DKK) also had the strongest effect of concussion on adverse transitions (HR 1.46, CI 1.34–1.58), relative to the other income categories.

Finally, individuals with concussion who were wage earners with management experience had the strongest effect of concussion on favourable transitions (HR 0.60, CI 0.45–0.81).

## Discussion

This study examined the long-term labour market consequences of concussion with a multistate model. The model enabled the analysis of labour market transitions using Danish administrative register data in a large cohort of hospital treated individuals with concussion compared to matched controls in relation to different socioeconomic and health characteristics.

As we hypothesized, this study found a lower incidence of favourable transitions, i.e., return to employment after a period of either sick leave, unemployment, or limited attachment to the labour market (e.g., flex job) among individuals with concussion compared to matched controls. Conversely, individuals with concussion also had a higher incidence of adverse transitions, i.e., transitioning to sick leave, unemployment, and limited attachment to the labour market after being employed or being on temporary leave.

Concussion is the mildest form of head trauma. In most cases it is associated with full spontaneous recovery during the first post-injury weeks. However, there is a growing body of evidence indicating that a part of the concussed population experience long-lasting consequences that also affect their ability to work. For instance, a substantial number of individuals with concussion are not working 12 months post-injury [30], and have higher odds of not attending ordinary work, long-term sickness absence and work disability, even 5 years post-injury compared to matched controls [10].

The results of this study show that the multistate model can provide new insight into which labour market states individuals with concussion transition to according to their incidence. This knowledge can guide management of the affected individuals across the different spheres of employment, health- and social care to prevent long-term sick leave and exclusion from the labour market. Research shows if recovery does not occur spontaneously, interdisciplinary, and vocational rehabilitation may reduce PCS and support work resumption [31, 32]. In Denmark national clinical guidelines describing the evidence for nonpharmacological interventions in adults experiencing PCS have been prepared to support clinical practice [33].

The reported results reflect the process from three months post-injury where we started registration of the different labour market states. This was to exclude the effects of the acute phase with a high incidence of initial sick leave and instead focus on the long-term effects of concussion as reflected in a wider range of social benefits that the patient starts receiving. Hence, the effects we see in this study after 3 months can be attributed to persisting symptoms after concussion and not the acute problems. For that reason, we believe our results extent the current research based on nationwide register studies. Further, the multistate model was able to capture the complexity of the Danish welfare system with the possible transitions between the labour market states and point at where in the trajectory the individuals might be most affected. In Denmark, in case of concussion-related sickness absence, unemployment or work disability, the affected person is ensured economic compensation with state-regulated social transfer payments depending on the duration of time spend off work. Our results in Table 2 indicate that sick leave is the most difficult labour market state to return to employment from compared to the other examined labour market transitions in this study, for both individuals with concussion and the background population. However, individuals with concussion are disproportionally more affected. The results are consistent with previous Danish research showing that individuals with concussion are more likely to receive health-related benefits including sickness benefit compared to the background population [10]. However, the Danish system is complicated and changes every few years in relation to legislation, and is difficult to generalise to other systems in other countries.

The results in this study only showed small differences between the subgroups of persons with concussion in relation to favourable and adverse labour market transitions. The results on age groups showed that ages between 30–39 years and 40–49 years had the lowest relative incidence of returning to work after concussion compared to controls. Additionally, although no significant difference in HR for adverse transitions between age groups was found, age groups between 30–39-years seemed the most affected. The low labour market attachment after concussion among these age groups has earlier been scientifically discussed and might be explained by the challenges of coping with large life events like establishing careers and building a family during these years [10].

Our results also indicate that concussion had the strongest impact on wage earners with management experience who had the lowest incidence of favorable transitions. This contrasts with a previous study that showed that professional/semiprofessional and management categories are occupations with more independence and opportunities for decision-making, which positively affect return to work after mild traumatic brain injury [34]. A systematic review also presents limited and conflicting results regarding workplace-related factors for returning to work in employees with acquired brain injury, including mild traumatic brain injuries. This review highlights that managers were more likely to retain their jobs compared to non-managers [35]. The conflicting results might be attributed to managers using their cognitive abilities to adapt to employee responsibilities, which can be challenging for a person with a concussion whose cognitive functions are affected [36]. Additionally, other influencing factors may include work-related stressors, high expectations from both the managers themselves and their surroundings, and occupational demands that require managers to step down from their positions or search for another job.

Higher income generally provides greater financial security [37], and makes it easier for individuals to take time off work for recovery and rehabilitation. However, this study found an inversed social gradient for income level, where individuals with PCS with high income had the greatest impact of concussion on both favourable and adverse transitions. Low income has previously been found to be associated with low employment rates following an injury (3 months post injury) [38], which is contrasting to the results of this study. Another contrasting study demonstrates that high income and high education in younger stroke survivores is associated with higher rates of return to work [39]. To our knowledge no previous studies have investigated the effect of social inequalities on labour market outcomes following concussion, why this could be prioritized in future research [40].

## Strengths and limitations

This study takes advantage of the availability of comprehensive, accurate and objectively measured data from nationwide registers which enabled us to examine a large representative population of individuals with concussion treated in Danish hospitals and matched to controls from the background population. This allowed for powerful statistical analyses and long-term follow-up.

Register data eliminate selection bias and provide valuable information on potential confounders. But not all information is accessible from registers in contrast to self-report data, and residual confounding should be considered [41]. Danish registers are generally considered of high validity. However, the ICD-10 diagnosis codes in DNPR may not be as accurate and consistent as the clinical diagnoses registered in patient records [42]. This may lead to misclassification and underestimation of the true incidence of concussion [43] and the exposure of labour market effects, beyond what is reported in this study. Also, we only had access to data from secondary care (hospital services, including emergency department visits and outpatient treatment), since no national registers of diagnoses registered by general practitioners can be accessed. This also contributes to uncertain estimates of the disease incidence and its long-term effects.

We have chosen adverse vs. favourable transitions in bundles as our primary analysis. Several of the transitions, e.g., sick leave to unemployment, are partly driven by legislation rather than by health and labour-related functionality. Additionally, some transitions do not indicate clear changes in labour market attachment, e.g., unemployment to sick leave, or employed to pension and are not investigated. Additionally, we did not distinguish between cases and controls working reduced hours or full time during sick leave and disability pension.

## Conclusion

Concussion may influence many aspects of life, including long-term employment. Opposite to earlier studies focusing on a single labour market state, this study examined all the possible transitions between labour market states an individual can experience, excluding the first three months post-injury. Individuals with concussion had more difficulties returning to the labour market following a period of e.g., sick leave or unemployment compared to the background population. These findings emphasize the importance of timely intervention, such as the accessibility to rehabilitation services for these individuals to prevent long-term work absence and exclusion from the labour market.

### Supplementary Information

**Additional file 1: eTable 1.** International Classification of Diseases, version 10 (ICD-10) diagnosis codes to describe inclusion- and exclusion criteria. This supplementary file contains information on the International Classification of Diseases, version 10 (ICD-10) diagnosis codes to describe inclusion- and exclusion criteria for included individuals with concussion and matched controls.

## Data Availability

The data that support the findings of this study are available from Statistics Denmark, but restrictions apply to the availability of these data, which were used under license for the current study, and so are not publicly available. Data are however available from the authors upon reasonable request and with permission of Statistics Denmark. Researchers interested in using the data for scientific purposes should contact corresponding author, Heidi Jeannet Graff, Email: heidi.graff@supermail.dk.

## Abbreviations

PCS: Persisting post-concussive symptoms
RTW: Return to work
CRS: Danish Civil Registration System
DNPR: Danish National Patient Register
ICD-10: International Classification of Diseases, version 10
DREAM: Danish Register for Evaluation of Marginalization
EoF: End-of-Follow-up
DSM-IV: Diagnostic and Statistical Manual of Mental Disorders, Fourth Edition
CCI: Charlson’s comorbidity index
