# Supplementary material for: Labour market attachment dynamics in patients with concussion: a Danish nationwide register-based cohort study
Source: BMC Public Health. 2023 Dec 13;23:2493. doi: 10.1186/s12889-023-17364-2 (PMC10717667; doi:10.1186/s12889-023-17364-2)
Supplement: Supplementary file 1 — Additional file 1: eTable 1. International Classification of Diseases, version 10 (ICD-10) diagnosis codes to describe inclusion- and exclusion criteria. This supplementary file contains information on the International Classification of Diseases, version 10 (ICD-10) diagnosis codes to describe inclusion- and exclusion criteria for included individuals with concussion and matched controls. [file 12889_2023_17364_MOESM1_ESM.docx]

**Additional file 1:**

eTable 1: International Classification of Diseases, version 10 (ICD-10) diagnosis codes to describe

inclusion- and exclusion criteria

| **Diagnosis** | **ICD-10 code** |
| --- | --- |
| ***Inclusion criteria (year 2003-2007) primary diagnosis*** |  |
| Concussion (Commotio cerebri) | S06.0 |
| ***Exclusion criteria (year 1998-2002) (2003-2007) primary and secondary diagnosis*** |  |
| Fracture of skull and facial bones  Fracture of neck  Dislocation, sprain and strain of joints and ligaments at neck level  Injury of nerves and spinal cord at neck level  Fracture of rib(s), sternum, and thoracic spine  Dislocation, sprain and strain of joints and ligaments of thorax  Injury of nerves and spinal cord at thorax level  Fracture of lumbar spine and pelvis  Dislocation, sprain and strain of joints and ligaments of lumbar spine and pelvis  Injury of nerves and lumbar spinal cord at abdomen, lower back, and pelvis level  Other injuries involving multiple body regions, not elsewhere classified | S12.0, S12.1, S12.2, S12.7,  S12.8, S13.0, S13.1, S14.0, S14.1, S22.0, S22.1, S23.1, S24.0, S24.1, S32.0, S32.1  S32.2, S32.7, S33.1, S34.0  S34.1, S34.3, T06.0, T06.1 |
| Intracranial injury  Crushing injury of head  Other and unspecified injuries of head  Crushing injuries involving multiple body regions  Other injuries involving multiple body regions, not elsewhere classified | S06.1-S06.3, S06.4-S06.6, S.06.7-S06.9, S07.0-S07.1, S09.7, T04.0, T06.0 |
| Concussion (Commotio cerebri) ***(1998-2002)*** | S06.0 |
